# Shape Optimization of Costal Cartilage Framework Fabrication Based on Finite Element Analysis for Reducing Incidence of Auricular Reconstruction Complications

**DOI:** 10.3389/fbioe.2021.766599

**Published:** 2021-12-13

**Authors:** Jing Zhong, Suijun Chen, Yanyan Zhao, Junfeiyang Yin, Yilin Wang, Haihuan Gong, Xueyuan Zhang, Jiejie Wang, Yaobin Wu, Wenhua Huang

**Affiliations:** ^1^ Guangdong Engineering Research Center for Translation of Medical 3D Printing Application, Guangdong Provincial Key Laboratory of Medical Biomechanics, National Key Discipline of Human Anatomy, School of Basic Medical Sciences, Southern Medical University, Guangzhou, China; ^2^ Department of Otolaryngology, Sun Yat-sen Memorial Hospital, Sun Yat-sen University, Guangzhou, China

**Keywords:** auricular reconstruction, skin necrosis, finite element analysis, shape optimization, complications

## Abstract

Skin necrosis is the most common complication in total auricular reconstruction, which is mainly induced by vascular compromise and local stress concentration of the overlying skin. Previous studies generally emphasized the increase in the skin flap blood supply, while few reports considered the mechanical factors. However, skin injury is inevitable due to uneasily altered loads generated by the intraoperative continuous negative suction and uneven cartilage framework structure. Herein, this study aims to attain the stable design protocol of the ear cartilage framework to decrease mechanical damage and the incidence of skin necrosis. Finite element analysis was initially utilized to simulate the reconstructive process while the shape optimization technique was then adopted to optimize the three-pretested shape of the hollows inside the scapha and fossa triangularis under negative suction pressure. Finally, the optimal results would be output automatically to meet clinical requirement. Guided by the results of FE-based shape optimization, the optimum framework with the smallest holes inside the scapha and fossa triangularis was derived. Subsequent finite element analysis results also demonstrated the displacement and stress of the post-optimized model were declined 64.9 and 40.1%, respectively. The following clinical study was performed to reveal that this new design reported lower rates of skin necrosis decrease to 5.08%, as well as the cartilage disclosure decreased sharply from 14.2 to 3.39% compared to the conventional method. Both the biomechanical analysis and the clinical study confirmed that the novel design framework could effectively reduce the rates of skin necrosis, which shows important clinical significance for protecting against skin necrosis.

## Introduction

Microtia is a congenital deformity characterized by the underdevelopment of the external ear, with an estimated annual incidence of up to 30.6 cases per 100,000 population in China (Deng et al., 2016). Auricular reconstruction continued to be the most challenging technique among plastic surgery, with the main difficulties of framework fabrication and emergence of various complications (Rocke et al., 2014; Frenzel, 2015). The most accepted protocol among surgeons worldwide is that the Nagata technique and its modifications typically used the ipsilateral rib cartilage to form the framework in the first-stage surgery (Bly et al., 2016). Irrespective of the kind of operation, the postoperational complications were inevitable (Fu et al., 2019). Skin necrosis is one of the most common complications, which mainly occurred after the first-stage modification. Previous studies had discovered that vascular compromise and local stress concentration of the overlying skin were the two main contributors to skin ischemia, even skin necrosis (Anghinoni et al., 2015; Cugno and Bulstrode, 2019). Nowadays, much evidence focused on how to provide an adequate blood supply of the skin flap, but only few considered the mechanical issues (Márquez-Gutiérrez et al., 2017; Olcott et al., 2019).

According to skin mechanical research studies, the most common failure tests of skin are tensile failure tests, piercing tests, and tearing tests (Tamura et al., 2014). Clinical investigation of auricular reconstruction confirmed the same opinion that negative pressure suction underneath the skin altered the tensile deformation, the pressure in the suture zone, and the osculatory friction of the skin (Bonilla, 2018; Yamada, 2018). All these variations result in a reduction in healing time and harmful skin-related biomechanical consequences such as dehiscence, granulation (Wilkes et al., 2012), ischemia (Huang et al., 2019), and even ulceration (Yamada et al., 2017). However, the mechanical interaction between the skin and framework during the auricular reconstruction for lower complications and better clinical performance remains unclear. Therefore, exploring the mechanical behaviors of the relative motion between the skin and cartilage framework has great significance in protecting against skin-related complications.

Finite element (FE) analysis is an effective computerized method that was widely approved in the biomechanical field, especially suitable for simulating non-linear materials under various loads, such as human skin tissue (Retel et al., 2001). A novel FE-based shape optimization technique appeared recently to improve the performance of a process or the design to obtain the maximum benefit from it. The transplant design in maxillofacial surgery had extensively adopted this novel technique for obtaining better function and performance (Shi et al., 2007; Luo et al., 2015). Rajabi et al. conducted a transposition flap FEA, which revealed the stress–stretch parameters in the process of wound closure by sutures (Rajabi et al., 2015). Li et al. proposed novel scaffolds with FE-based shape optimization that provided a recommendable design protocol in tooth root reconstruction (Li et al., 2010). In auricular plasty, Miyamoto and Nagasao et al. evaluated the diverse otoplasties in the prominent ear with FE methods to advance insights into this field and give a suggestion with a better surgical protocol (Miyamoto et al., 2009; Nagasao et al., 2014). Jiang et al. researched the thickness parameter of the auricular silicone scaffold with FEA that finally obtained an optimized thickness with sufficient intensity and hardness (Jiang et al., 2016). Therefore, it is feasible to develop an FE-based shape optimization to determine the mechanical behavior of the skin and the optimum auricular structure. Based on preceding studies, several efficacious approaches had been discovered that includes alteration of suture materials (Sakamoto et al., 2012), stability of ear framework (Mazeed et al., 2021), and decrease in the span using the drain to reduce skin stress and necrosis (Bangun et al., 2010). However, these methods mainly relied on the clinical experiences of surgeons, which is a lack of related mechanical study to elucidate the relationship among the ear framework structure, suture, and skin necrosis.

**SCHEME 1:**
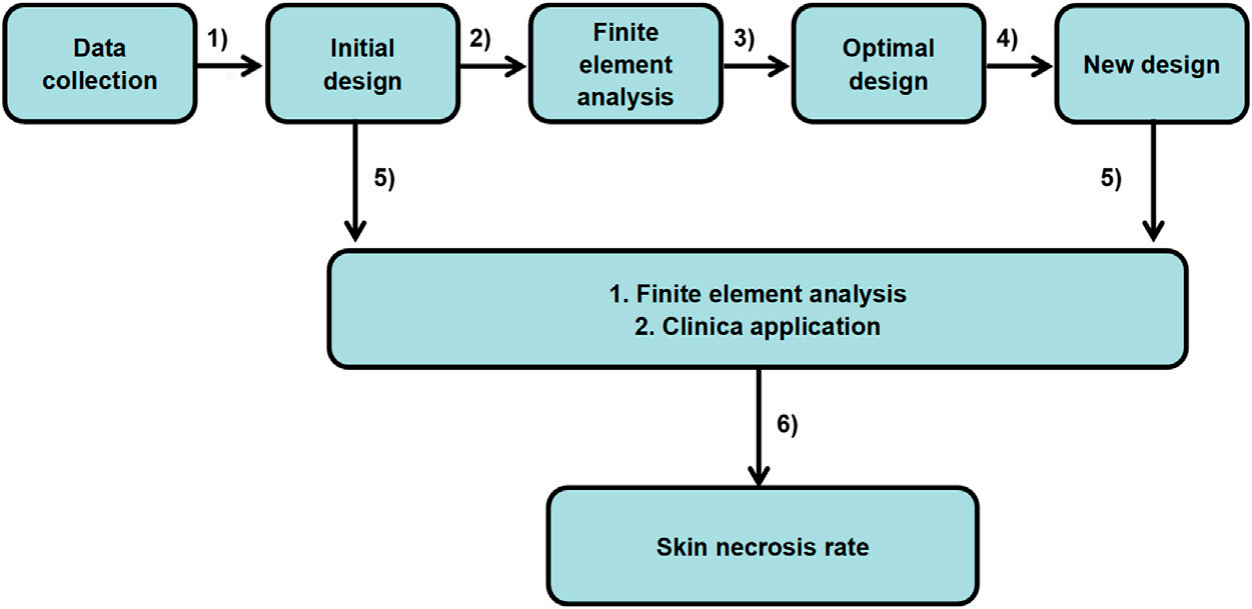
Optimal design of the ear cartilage framework flowchart: from patient data to clinical application.

Herein, we present a design protocol of the ear cartilage framework to decrease mechanical damage and incidence of skin necrosis, based on biomechanical research in skin necrosis (Scheme 1). Finite element analysis was initially utilized to simulate the reconstructive process while the shape optimization technique was then adopted to optimize the three-pretested shape of the hollows inside the scapha and fossa triangularis under negative suction pressure. Finally, the optimal results would be output automatically to meet clinical requirement. Guided by the results of FE-based shape optimization, the optimum framework with the smallest holes inside the scapha and fossa triangularis was derived. The biomechanical analysis had confirmed that the stable framework structure could effectively decrease the rates of mechanical injury under the continuous negative pressure loading. And the clinical survey also showed the optimized ear framework could efficaciously reduce the incidence of skin necrosis compared to the conventionally fabricated framework. Our study creatively established a computerized FE model to mimic the process of the first-stage reconstruction during the negative suction period and also proposed a novel design method with shape optimization to reduce skin-related postoperational complications. This study provides advanced insights into biomechanical behaviors in auricular reconstruction, offers a reference for the surgeons, and achieves important clinical significance for protecting against skin necrosis.

## Materials and Methods

### Data Acquisition and Processing

**FIGURE 1:**
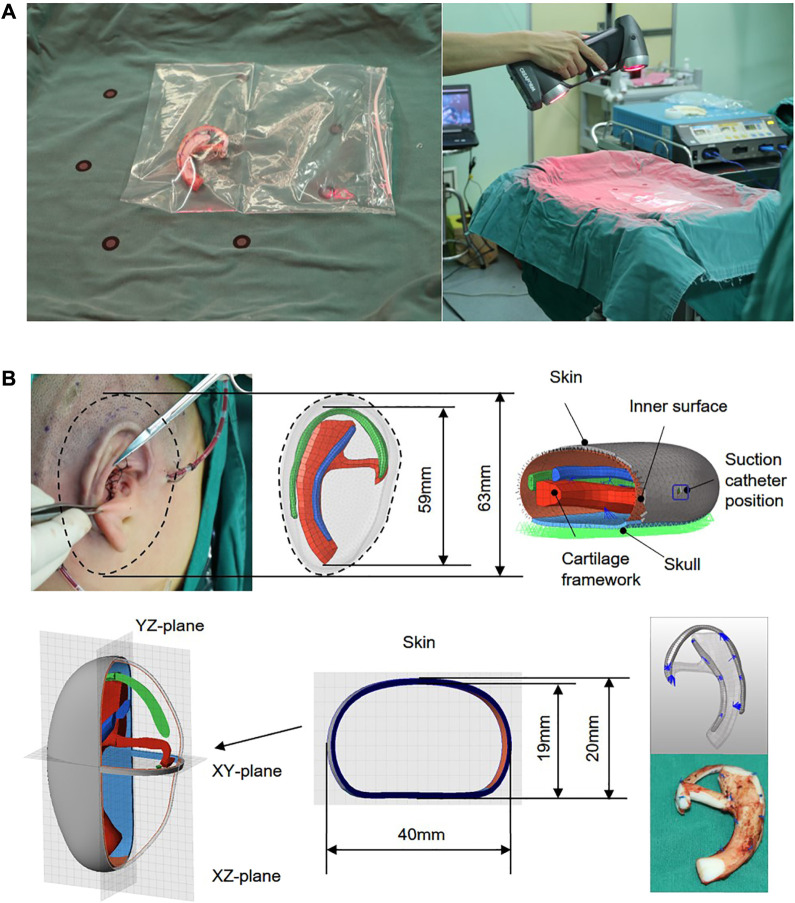
Autogenous costal cartilage ear framework and captured the 3D images of the cartilage framework by a laser scan **(A)**. CAD modeling and definition of models used for finite element analysis. A vacuum suction loading condition of 0.01 MPa (75 mmHg) was applied onto the inner surface of the skin. The framework was placed in the skull, and the boundary conditions of a skull design were set as fixed. Quadrilateral symbol indicates the suction catheter position, a 0.5-mm-wide incision cut from the skin surface to the void, and all blue sutures of the FEA model are rigidly fixed by using the simulated surgical method **(B)**.

A 7-year-old volunteer with grade III of the right side of the congenital microtia was recruited with written informed consent. Study procedures were approved by the Committee of Sun Yat-Sen Memorial Hospital. In the first stage, the synchondrosis between the sixth and seventh costal cartilages, and the eighth and ninth costal cartilages was harvested and then carved to model the base frame, upper helix, and antihelix of the ear framework. All the details regarding the design are summarized in Table 1. Three parts of the ear framework were constructed with absorbable sutures to be generated as a whole. The intact ear framework was wrapped in a transparent aseptic bag to place on an operating table, and then the three-dimensional (3D) images of the cartilage scaffold were captured by a laser scanner (Handscan700, Creaform, Canada). During the 3D scanning process, the scanner should be kept away from the sterile operating table (Figure 1A).

**TABLE 1:** Cartilage fabrication details of the ear framework.

Assembly	Thickness (mm)	Cartilaginous harvesting
Base frame	5	The synchondrosis between the sixth and seventh costal cartilages
Upper helix	5	The ninth costal cartilages
Antihelix	3	The eighth costal cartilages

### Geometry Reconstruction

The 3D models of ear framework and covering skin were created and obtained separately using Geomagic Freeform software and the Touch X Haptic (3D Systems, Rock Hill, SC) (Figure 1B). Subsequently, the whole geometric model, which contains cartilage frame, skull, and the skin (1 mm), was imported into FEA preprocessing software Hypermesh (HyperWorks 14.0, Altair, United States) for meshing and set of loading constraints, contact conditions, and friction coefficient. Since the vacuuming process mainly focused on the interaction between the covering skin and the framework after contact, and the process without their interaction was not the main consideration, we simulated the vacuuming process with a minimum distance of 2 mm between the skin and the superior edge of the upper helix of the framework.

### Auricular Reconstructed Transplant FEA

Radioss (HyperWorks 14.0, Altair, United States) was used to simulate the transplantation process of the first-stage ear reconstruction surgery. The covering skin was defined as non-linear hyperelastic material by an Ogden hyperelastic model (Shergold and Fleck, 2005; Delalleau et al., 2006). The auricula framework and skull were defined as a linear plastic material and isotropic material (Griffin et al., 2020; Zhao and Narwani, 2005; Motherway et al., 2009). Table 2 summarizes the material properties used for FEA. The framework was placed on the skull, and the boundary conditions of a skull design were set as fixed. The interface TYPE7 is a general purpose interface with penetration preventing the node from going through the shell mid-surface. And it can also simulate all types of impact between a set of nodes and a master surface. Therefore, we chose the self-contacted interaction between the skin and ear framework to be set as interface TYPE7 by using the non-linear penalty function. Meanwhile, the minimum contact gap (Gap_min_) was set as 0.09 for reducing the penetration by allowing nodes to slow down over a larger distance. The friction coefficient between the skin and ear framework was set as 0.3 (Cua et al., 1990). Meanwhile, a vacuum suction loading condition of 0.01 MPa (75 mmHg) was applied onto the inner surface of the skin (refer to the negative pressure value of the intraoperative drainage device), and the loading subject was continuously applied to the shell element of the inner skin in a vertical direction. To simulate the vacuuming process and reduce the influence of dynamic disturbance, the loading was applied stepwise. At time 0, no loads were applied to the model. The skin remained in its original drum-like shape, and the framework was inside the skin. From 0 to 0.5 s, the pressure was linearly added to 0.01 MPa onto the inner surface of the skin. Starting at 0.5 s, the negative pressure remained at 0.01 MPa (75 mmHg) up to 1 s, and model responses were observed.

**TABLE 2:** Finite element analysis computer model: material properties.

Tissue	Model	Density (g/cm^3^)	Elasticity modulus (MPa)	Poisson’s ratio
Human skin	Ogden hyperelastic	1.18	0.86	0.46
Costal cartilage	Linear	1.11	11.43	0.4
Skull	Linear	1.92	15,000	0.3

### Optimization Routine

**FIGURE 2:**
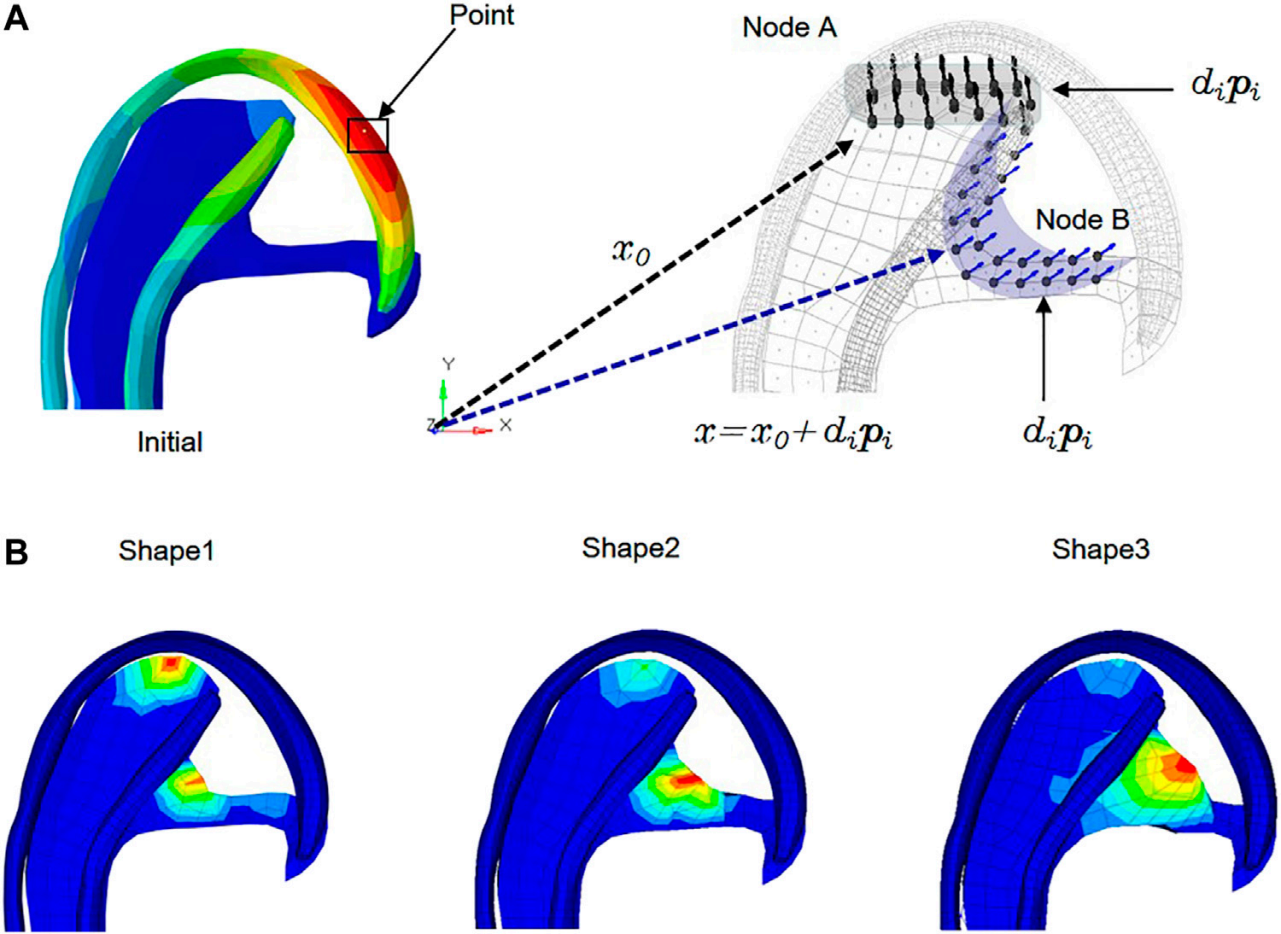
Allowable movement of the control nodes set up in the finite element model. The arrows indicate the direction of allowable movement. The control nodes were moved along vectors adjacent to the surface normal in the shape optimization analysis. Following each optimization iteration, the finite element model was reconfigured such that nodes on the 3D framework were made coincident with the adjusted control nodes. X_0_: Vector of nodal coordinates in the initial design. Pi: Defines the changes of nodal locations with respect to the original finite element mesh. Di: Design variable. *X* = *x*_0_+dipi: final location. The shaded areas indicate the deformable areas **(A)**. The shape of three design variables at the iteration number of 6, 7, and 6 reached convergences, respectively **(B)**.

The stress-regulated shape change of the costal cartilage framework was predicted by the auricular reconstructed transplant process simulation by numeric optimization in conjunction with a finite element analysis. Changes in the design parameters were used to alter the shape of the finite element model and produce a new design. The framework shown in Figure 2 was pre-deformation using the “HyperMorph” function in the shape optimization method to reduce the deformation concentration around its helix. Summing the contributions to design node movement due to the influence of single shape variables to a new mesh was generated moving in the direction of the arrow, and the design variables of the node are shown in Table 3. In three pretests, the yellow node of deformation constraint was reduced by 5, 7, and 15%, respectively. In order to ensure the minimum deformation and unidirectional growth of the ear framework during the vacuuming process, the minimizing structure compliance was equivalent to maximum stiffness as the objective function, and the shape of the ear was optimized with OptiStruct (HyperWorks 14.0, Altair, United States). According to the results of each iteration, the nodes automatically generated more grids along the arrow direction, and the change in the grid number automatically reflected in the shape of the ear framework until the target converged and the iteration ended, and then the shape optimization was completed successfully.

**TABLE 3:** Design variables of nodes.

Define shape	Shape 1		Shape 2		Shape 3	
Desvar	Node A	Node B	Node A	Node B	Node A	Node B
Initial value	0	0	0	0	0	0
Lower bound	0	0	0	0	0	0
Upper bound	1.5	1.2	1.5	2.0	1.1	1.1

### Mechanical Experiment Validation

Even though FE analysis could provide very useful numerical results for evaluating the effectiveness of the optimized design, the experimental proof was still necessary. Therefore, mechanical tests were performed to compare the skin surface strain field in the initial and optimized framework. Due to the complexity of the framework structure, manual carving techniques cannot accurately restore the model structure. Therefore, we chose 3D printing for modeling and assigned the 3D printing material parameters to the finite element model. The postauricular skin with a diameter of 8 mm was taken from a fresh corpse and then connected to the base, which was maintained in airtight containers.

**FIGURE 3:**
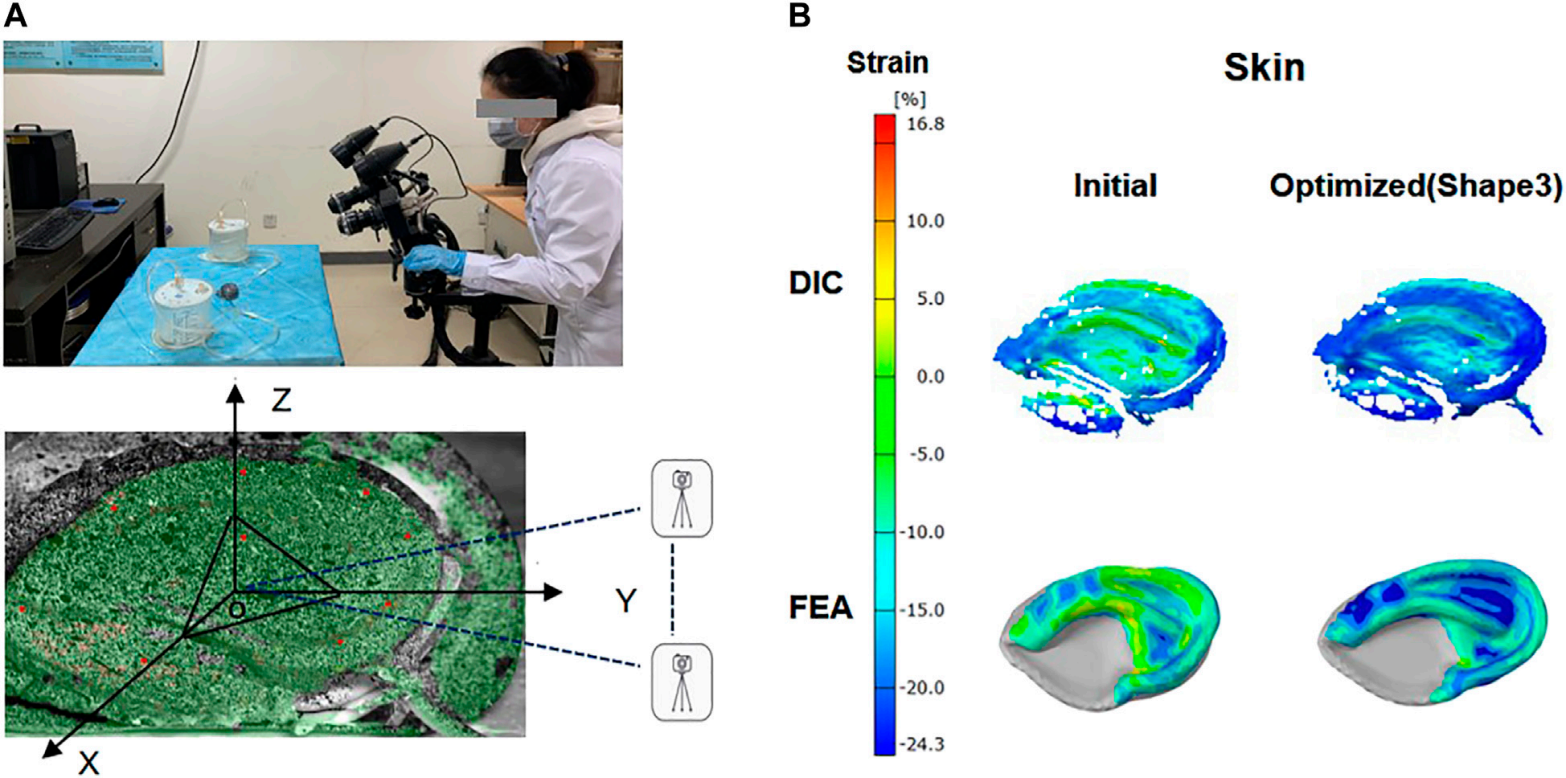
Digital image correlation (DIC) system and ear framework model covered by skin. The anterior surfaces of skin were painted with spray paint for DIC with white background color and a black random speckle pattern **(A)**. Contour plot: skin surface strain field in the initial and optimized model (shape 3) was obtained by FEA analysis and DIC measurements **(B)**.

The suction catheter was placed in the space between the skin and the framework in accordance with the operation, and the skin was drawn onto the framework with negative pressure. During mechanical testing, the anterior surfaces of the covering skin strain were recorded using DIC (ARAMIS 4M, GOM, Germany) as previously reported (Sutton, 2008). To better collect data, the model was placed in the X-O-Y plane, and the bottom of the outer edge was raised 30° along the Z-axis. Two digital cameras with a frame rate of 4 Hz were placed along the Y-axis, and a red light source was distributed on the surface of the skin as far as possible to create a visual 3D image (Figure 3). Then negative pressure continuous suction was used to maintain a vacuum condition, and images for calculation of skin surface strain were obtained by DIC when the vacuuming process lasted for 1 s. According to the aforementioned method, the initial and optimized models were tested, respectively, to extract the strain contour plots of the clinically vulnerable areas.

### Patients and Follow-up

A retrospective review was conducted to collect data from inpatients who underwent autologous cartilage microtia reconstruction in the Department of Otolaryngology at the Sun Yat-sen Memorial Hospital of Sun Yat-sen University from January 2017 to June 2020. The inclusion criterion was the patients with grade III of congenital microtia underwent the modified Nagata technique with the first stage. All of these surgeries were performed by Suijun Chen, who has more than 7 years of ear reconstruction surgery experience. And all the included cases had been followed up for more than 3 months after the operation with patient data and a follow-up report. Patient data included the surgical method, the type of soft tissue coverage, ear framework structure, and postoperative removal of suction catheter time. And follow-up records included skin necrosis, framework exposure, infection, hematoma, and scars.

### Statistical Methods

The results of shape optimization were recorded, and the normality and the homogeneity of variance were primarily analyzed using Levene’s test. Data comparisons were conducted using a two-way analysis of variance (ANOVA). All statistical analyses were performed using IBM SPSS statistical software (IBM SPSS version 20). *p* < 0.05 was considered to indicate a statistically significant difference.

## Results

### Finite Element Analysis

**FIGURE 4:**
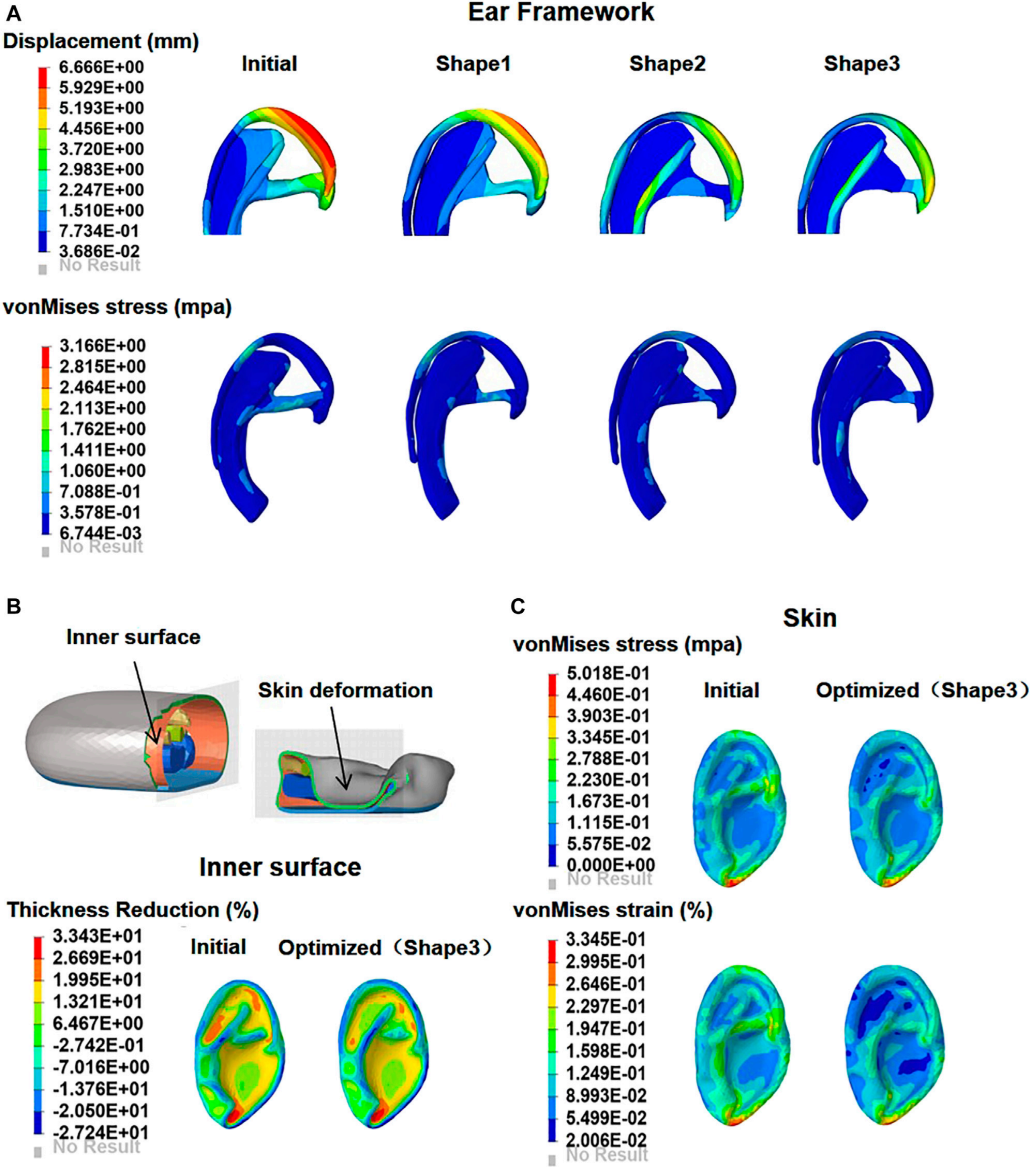
Contour plots of deformation and maximum von Mises stress in the initial and optimized models (shape 1, shape 2, and shape 3) **(A)**. The skin is drawn onto the framework as negative pressure is applied; comparison of the skin thinning rate distribution on the inner surface between initial and optimized model **(B)**. Contour plots of skin maximum von Mises stress and strain in the initial and optimized models (shape 3) **(C)**.

**FIGURE 5:**
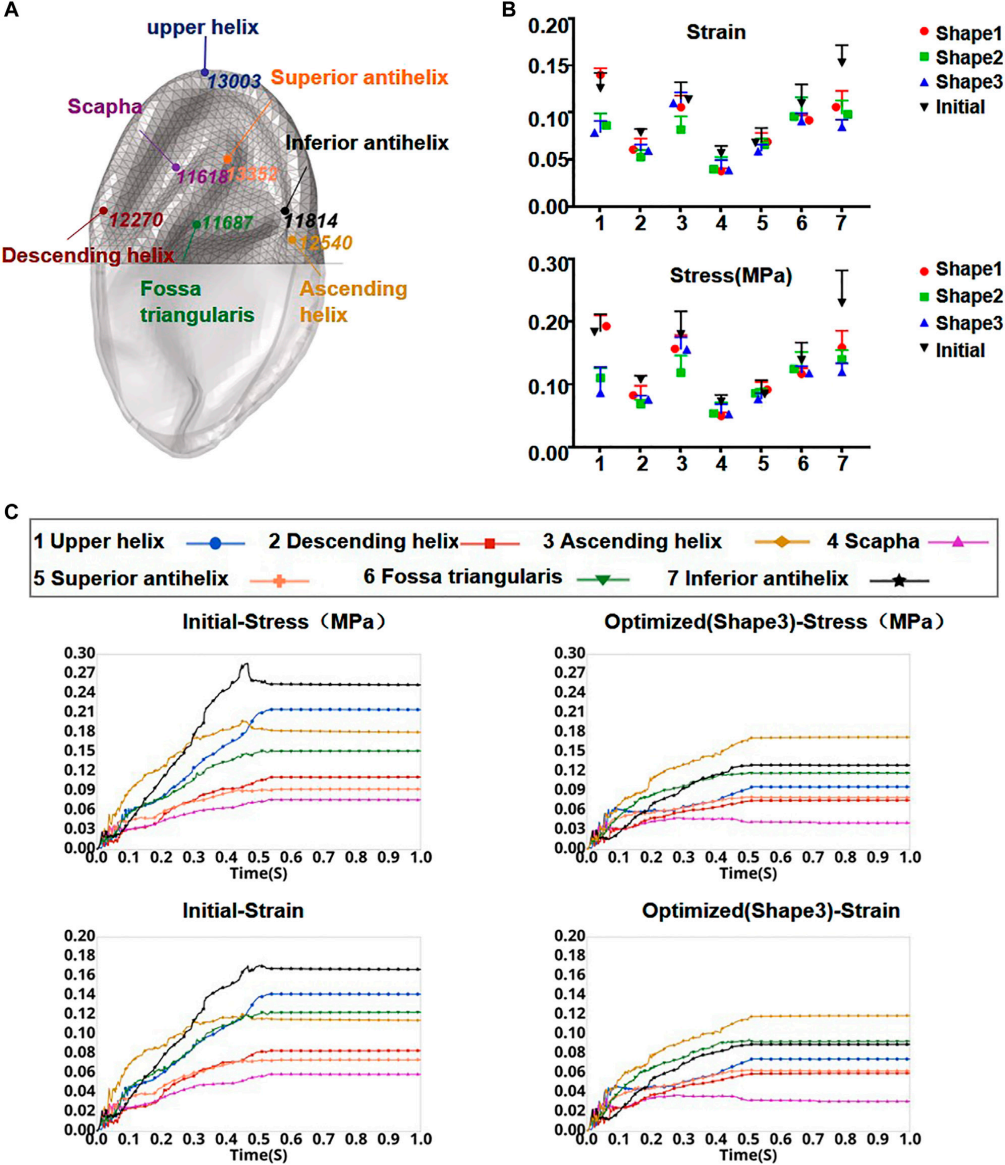
Element node numbers and locations in the skin observation area **(A)**. Stress and strain of seven locations from the initial model and each optimized model when the press was added to 0.01 MPa onto the inner surface of the skin **(B)**. Curves of skin strain and stress before and after optimization (shape 3) as a function of time in the observation area **(C)**.

According to the computation checks of FEA, Supplementary Figure S4 showed that the total energy in our model was equaled to the internal energy, kinematic energy, hourglass energy, and contact energy, with the total hourglass being only 1% of the total energy. These results verified that the whole simulation process respected the fundamental energy conservation laws. The FEA results of the one second were captured, including the total displacement and von Mises stress of the ear framework, as well as the thinning rate, von Mises stress and strain distributions of the covering skin (Figure 4, initial). The time–stress and time–strain curves of the covering skin at seven locations in the observation area are shown in Figure 5 (initial). The displacement of the framework was mainly concentrated over the helix, with a maximum value of 6.67 mm, while the von Mises stress areas were mainly concentrated at the suture point, with a maximum value of 3.17 MPa. The thinning parts of the covering skin were mainly concentrated in the antitragus, the scapha, and the triangular fossa, while the strain and stress were mainly concentrated in the antitragus, descending helix, and antihelix.

### Optimization and Further FEA

The numeric shape optimization was completed successfully, and the three design iterations reached 6, 7, and 6 converged, respectively **(** Supplementary Figures S1–S3 **)**. Ear framework outlines of the initial model and the optimal model are shown in Figure 2B. The maximum von Mises stress and total deformation of three optimized structures were decreased compared to FEA (Table 4). Among them, shape 3 showed the best stability under the same mechanical load. In addition, effectiveness in reducing the skin stress and strain in auricular reconstructed transplant processing of each model was also evaluated using further FEA (Figures 5A,B). Since the mechanical load in the skins was the same, we selected seven nodes with the same ID number in the skin elements for statistical comparisons. These results showed that the skin stress and strain of two optimized (shape 2 and shape 3) structures were significantly different from those of the initial model (*p* < 0.05), and there was no statistical difference between shape 1 and the initial model (*p* > 0.05). Therefore, shape 3 with the best stability was chosen as the optimal result and compared with the initial framework. Results showed that the thickness reduction, von Mises stress, and strain of shape 3 were all decreased (Figures 4B, C). The detailed analysis of the seven nodes with the same ID number showed that the stress and strain of shape 3 decreased significantly, with a stress reduction range from 9.41 to 52.7% and a strain reduction range from 3.51 to 44.4% (Figure 5C and Table 5).

**TABLE 4:** Values of initial and optimized frameworks of total deformation and von Mises stress under the same mechanical load.

Framework	Total deformation (mm)	von Mises stress (MPa)
Initial	6.67	3.17
Shape 1	6.39	2.34
Shape 2	3.80	2.29
Shape 3	2.34	1.90

**TABLE 5:** Initial values, optimized (shape 3) values, and reduction proportion of strain and von Mises stress (MPa) in the whole model and respective components.

	Skin stress (MPa)			Skin strain		
	Initial	Optimized	Reduction	Initial	Optimized	Reduction
Upper helix	0.184	0.087	52.7%	0.126	0.079	37.3%
Ascending helix	0.108	0.076	29.6%	0.079	0.060	24.1%
Descending helix	0.180	0.156	13.3%	0.114	0.110	3.51%
Scapha	0.073	0.053	27.4%	0.057	0.039	31.6%
Superior antihelix	0.085	0.077	9.41%	0.068	0.059	13.2%
Fossa triangularis	0.139	0.118	15.1%	0.110	0.091	17.3%
Inferior antihelix	0.230	0.120	47.8%	0.153	0.085	44.4%

### DIC Image Analysis

The skin surface strain behavior to cover the ear framework is shown in Figure 3. According to the DIC analysis results, the strain in the observation area of the initial model was mainly concentrated on the superior edge of the upper helix and the inferior crus of the antihelix in the vacuuming process, and the skin strain in the observation area after optimization was significantly reduced. These results coincided well with the skin surface strain results of the FEA.

### Complications

According to the inclusion criteria, the effective postoperative follow-up records of 216 patients were archived, of which the ear framework of 98 patients was not filled up in the scapha and fossa triangularis, and the ear framework of 118 patients was filled up in the scapha and fossa triangularis. Among 98 patients, infection of the new ear was observed in three patients (3.06%), the subcutaneous hematoma was observed in four patients (4.08%), the hypertrophic scar was developed in four patients (4.08%), skin necrosis was detected in 16 patients (16.3%), and cartilage exposure occurred in 13 patients (14.2%). However, this new design reported lower rates of skin necrosis from 16.3% decreased to 5.08%, as well as the cartilage disclosure decreased sharply from 14.2 to 3.39% compared to the conventional method (Table 6).

**TABLE 6:** Complications after surgical first-stage procedures.

	Not filled up	Filled up	Total
	(*N* = 98), n (%)	(*N* = 118), n (%)	(*N* = 216), n (%)
Skin necrosis	16 (16.3)	6 (5.08)	22 (10.2)
Exposure	13 (14.2)	4 (3.39)	17 (7.87)
Infection	3 (3.06)	3 (2.54)	6 (2.78)
Hypertrophic scar	4 (4.08)	4 (3.39)	8 (3.70)
Hematoma	4 (4.08)	2 (1.69)	6 (2.78)

## Discussion

Total auricular reconstruction is a challenging procedure that is generally performed in three stages, while the primary phase is the key to achieve the optimal outcome (Yamada, 2018). However, skin necrosis is one of the most common complications, of which the majority occurred in the first-stage surgery to require immediate attention and appropriate management (Anghinoni et al., 2015; Cugno and Bulstrode, 2019). Previous evidence discovered that vascular compromise and local stress concentration of the overlying skin were the two main contributors to skin ischemia and cartilage exposure. Continuous studies had reported various aspects for providing an adequate blood supply of the skin flap, but few reports have focused on the mechanical factors. During the first surgical phase, it is necessary to place drains with a negative suction vacuum pressure of 0.01 Mpa (Sasaki et al., 2021) between the skin envelop and underlying cartilage for 3–7 days (Bonilla, 2018), which for obtaining a hermetic closure of the tissues to achieve adequate adhesion of the tissue with a better ear form (Torres et al., 2021). However, the sustaining suction would create a negative press at the inner surface of the skin flap, which produces the passive stretch tension inside the skin. Meanwhile, the friction and pressure forces were spontaneously generated to resist the relative motion between the skin and the unfixed structure of the cartilage framework during the negative suction period, with the exception of the cartilage bottom and the surface of the skull. The aforementioned forces generated by negative suction probably cause skin failure and then increase the risks of skin necrosis (Walton and Beahm, 2002; Jing and Hong-Xing, 2007). Therefore, we believe that it is worthy information regarding the skin’s mechanical behavior and deformation properties during the suction process, which is critical for improving the clinical decision to decrease the focused stress and the rates of skin problems. Abundant research studies indicated that the stable support underneath the skin could disperse the region with high stress and thus decrease the risk of skin necrosis (Wilkes et al., 2012; Tamura et al., 2014; Mazeed et al., 2021) but not reported in the auricular reconstruction field. Zhang et al. proposed a novel concept of three-dimensional mechanical equilibrium cartilage scaffold, which could obtain a more stable structure by achieving lower rates of skin complications (Zhang et al., 2019). However, their research was limited to clinical investigation, without advances in mechanical study to explore the deep connections between the structural mechanical properties and skin necrosis.

FEA is an effective tool for quantitatively assessing regional differences in the mechanical properties of soft tissues (Roth et al., 2013). It is not only available to obtain the stress–strain distribution of non-linear materials (such as skin tissue) and also could be assessed the biomechanical response of soft tissues after stress application, especially the structural design evaluation of implants (Xu et al., 2020). A three-dimensional scanner was adopted in this study to obtain the digital three-dimensional (3D) model of the autologous costal cartilage ear framework, which was harvested and carved with the modified Nagata technique (Bly et al., 2016). Then this 3D model was imported into the dynamic FEA platform to mimic the whole negative suction process in the first-stage modifications, which displays the mechanical properties of the skin and cartilage separately. After applying a 0.01 MPa negative pressure for one second, the dynamic FEA results showed the most significant deformation occurred at ascending helix, while the stress concentration was located at the sutures zone (Figure 4A). Furthermore, the overlying skin graft would gradually tend to adhere steadily to the cartilage from 0.5 to 1 s, as well as the FEA results discovered the maximal stress and strain area were situated at the lobule, and ascending helix combined with the most thinning area of the skin was the lobule, scapha, and fossa triangularis. Therefore, it is reasonable to assume that the location of ascending helix, suture zone, and the upper helix should pay more attention and require further improvement.

Sakamoto et al. presented the same opinion that the sutures buried inside the cartilage were susceptible to exposure at the surface by tearing the cartilage and skin (Sakamoto et al., 2012). Several clinical investigations of auricular reconstruction also demonstrated the skin necrosis typically observed at the helix after first-stage modification, which is consistent with our dynamic FEA results (Kim et al., 2017; Cugno and Bulstrode, 2019). The digital image correlation (DIC) test (Maiti et al., 2016; Xie et al., 2020) was conducted simultaneously to verify the validity of FEA models; due to the complex three-dimensional structure of ear cartilage, the areas of the concha and lobule were absent during the acquisition process with the DIC system. Therefore, the relatively flat locations of the whole ear cartilage in the DIC measurements were selected to compare with the same areas of the FEA contour plot. The stress distributions of the skin showed remarkably similar results of dynamic FEA, which could also confirm our FEA models are practical and feasible.

Our study creatively established a novel FEA model to mimic the process of the first-stage reconstruction, which could veritably observe the mechanical changes of the skin and cartilage under the negative suction stress. It also supported the surgery to survey the mechanical behaviors of cartilage underneath the skin directly. Hence, it is worthy of exploring the optimal structure of the costal cartilage ear framework to earn better stabilization with minimal stress/strain under continuous suction pressure. Based on the modified Nagata technique and the clinical experiences, the helix, antihelix, scapha, and fossa triangularis are essential to maintain the base frame of the auricular morphological features that their fabrication had generally reached a consensus among the global surgeons (Gandy et al., 2016; Wang et al., 2016). All these structures typically were sculpted as the reference with the parental or contralateral healthy ears (Li et al., 2018). Nevertheless, controversies remain among the surgeons regarding the sizes of the distinct hollows inside the scapha and fossa triangularis. Some scholars believed that larger distinct hollows were beneficial to attain favorable aesthetics with better anatomical details (Zhang et al., 2021; Yang et al., 2018). In contrast, others considered smaller cavities were conducive to stabilization of the cartilage framework (Bonilla, 2018). To obtain an accurate conclusion about the carving sizes of distinct hollows, we utilized the shape optimization approach to pretest the cavities of the scapha and fossa triangularis separately and then achieved three post-optimized models. Subsequently, three models were imported to the FEA platform for additional evaluation of the mechanical properties between the overlying skin and cartilage framework, in which the optimum one could guide the clinical design of these locations. The FEA results demonstrated all three post-optimized models were more stable than the original larger cavities of the scapha and fossa triangularis (Table 4). Previous clinical studies showed similar results. Li et al. presented their 10 years of surgical experience that the cavity inside the fossa triangularis was filled up to decrease the rates of skin necrosis due to the excessive protrude of the antihelix (Li et al., 2018). Meanwhile, Yamada (Yamada, 2018) and Fu et al. (Fu et al., 2019) advocated that the smaller hollows of scapha could increase the stabilization of the whole cartilage framework.

**FIGURE 6:**
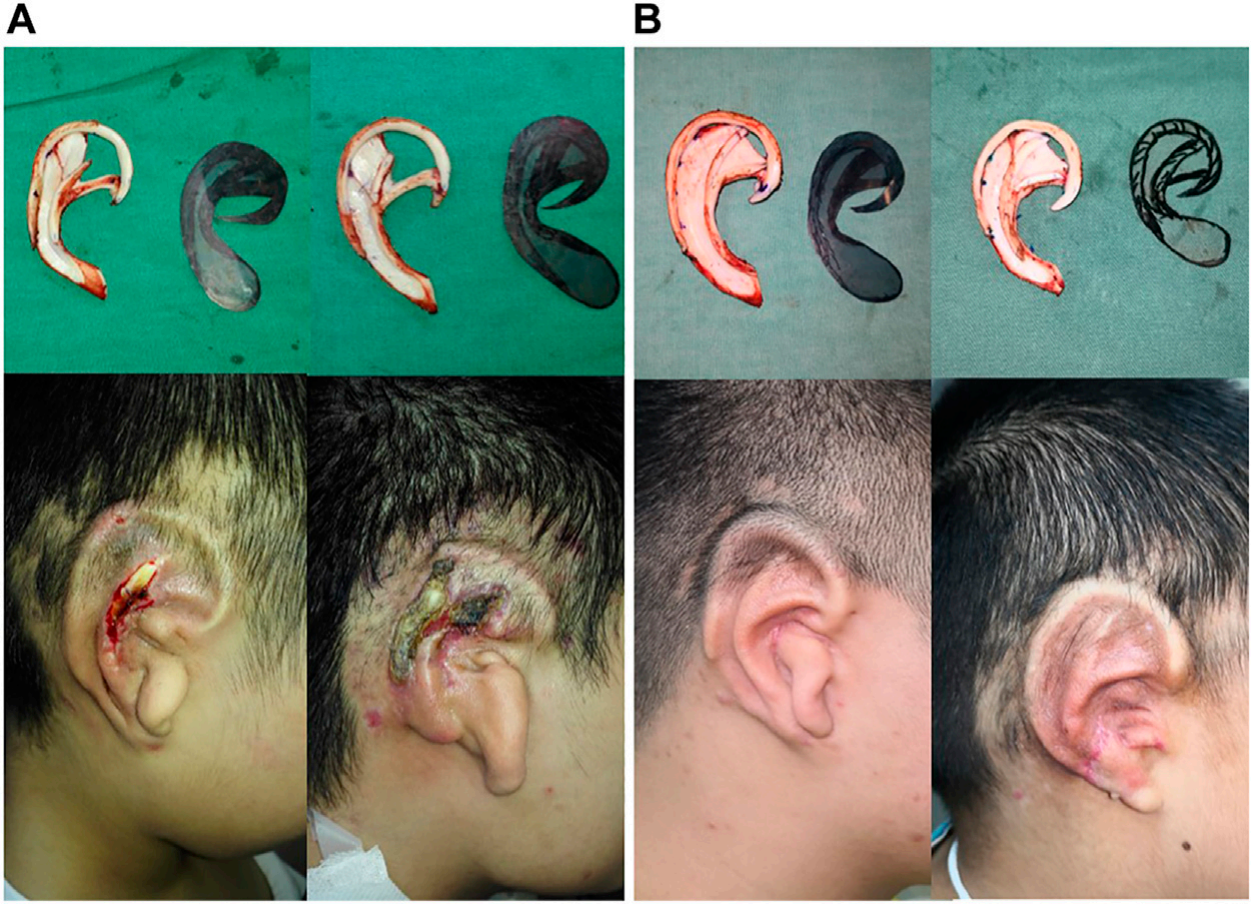
Auricle reconstruction using an autogenous cartilage framework in the first stage of the surgery. Regional skin necrosis of framework which did not filled up the scapha and fossa triangularis in 3 months **(A)**. Favorable postoperative results with a good definition of the auricular morphology without skin necrosis after 3 months occurred in the modified implanted ear framework, sculpted in filling up scapha and fossa triangularis **(B)**.

Based on the results of optimization analysis, the optimal structural design should fill up all the hollows inside scapha and fossa triangularis for effectively decreasing the stress and strain of the covering skin tissue. Furthermore, both the second shape and third shape models could smooth the over-prominent structures of the cartilage framework, as well as the stress and displacement of the third model cartilage were decreased markedly. There seem to be fewer holes of the scapha and fossa triangularis that could give more support to the helix and antihelix with favorable stability. Compared to the original model with unfilled hollows, the skin thinning ratio and the concentrated stress of the scapha and fossa triangularis declined dramatically. Besides, it is indicated that the more the filled area of the hollows, the lower will be the rates of skin damage at the upper helix, inferior antihelix, and scapha. All these sites were precisely the same areas which are susceptible to skin necrosis in the first-stage surgical phase. Since the simulation focus was on stage I of the operation, the lobule sculpture was mainly completed in the second and third stages. Therefore, the incidences of skin necrosis in this area are not taken into consideration. According to our biomechanical results, our team presented an optimal method of fabricating the cartilaginous ear framework as follows: 1. combining the separately fabricated ascending and inferior antihelix into integrated antihelix cartilage complex for achieving the smooth transition between the antihelix and baseplate; 2. carving bilateral slope of the superior crus of antihelix smoothly to decrease the risks of regional skin injury; 3. filling the hollows of the scapha and fossa triangularis as much as possible under the circumstance of sufficient cartilage to stable the whole ear cartilage framework, and also avoiding unevenly with the surrounding structure to decrease the local stress of prominent areas; 4. the fixed wire suture should not be too tight to increase unnecessary stress concentration. Therefore, the number of fixed sutures should be reduced as possible and widen the distance between the sutures to disperse local stress. Depending on the development of the cartilaginous ear framework, we performed a retrospective review (Fu et al., 2019) of consecutive patients *via* this optimal method or traditional ways with a follow-up period of 3 months to compare the rates of complications after first-stage modification. Because cartilage disclosure usually occurred after the skin injury, it could utilize as a secondary measurement (Cugno and Bulstrode, 2019). Compared to the traditional method, our novel optimum design reported lower rates of skin necrosis according to the same surgeon (from 16.3% decrease to 5.08%), as well as the cartilage disclosure decreased sharply from 14.2 to 3.39%. These clinical follow-up results once again confirm the validity and reasonableness of our dynamic FEA analysis and shape the optimization approach (Figure 6).

`There is an internationally accepted standard to evaluate outcomes in auricular reconstruction whether it could present a better aesthetic appearance, precise contour, and skin scarring after the surgery (Soukup et al., 2012). Nevertheless, the skin necrosis and framework leakage easily resulted in poor appearance, psychological stress, and even delaying the patient’s overall recovery. Based on our biomechanical analysis and clinical study, we considered the stability of the ear framework could be the independent risk factor for the complication after the surgery. Therefore, it is necessary to make a plan before the surgery by assessing the stability of the ear framework *via* FEA and relevant modification, which subsequently decreases the incidence of skin necrosis and increases the patient satisfaction (Reinisch et al., 2020).

There are several limitations to our study: the first is that we only analyzed the mechanical action of the ear framework and suture on the covering skin without considering other factors, such as the skin thickness, negative suction value, and the incision location. Further simulation models in this study should include more mechanical parameters and operating conditions to obtain a favorable function of the ear cartilage framework and a more elaborate appearance. The most noteworthy disadvantage of this study is its retrospective nature, and further clinical research is also necessary to improve credibility in a larger sample size with a multicenter, random, and open-control design.

## Conclusion

In summary, we presented the stable ear cartilage framework could effectively reduce the mechanical injury of the skin under the continuous negative pressure load. The results of the FEA and shape optimization approach indicated that filling up of the hollows inside the scapha and fossa triangularis was a benefit for increasing the stability of the whole ear cartilage framework. Compared to the conventional cartilage framework, we discovered the stress and strain in the susceptible area to skin problems were dispersed markedly after shape optimization design under equal conditions. Our clinical study also shared a similar outcome that the incidence of skin necrosis and cartilage exposure was decreased significantly with our novel approach. This may be the first research that utilized the dynamic FEA method to stimulate the first-stage transplant process, and we successfully established an effective digital model for further biomechanical study. It was demonstrated that the novel cartilage framework design was capable of reducing the relevant complications with proving *via* FEA and clinical evaluation, which could give accurate reference for the ear reconstruction surgeons. To solve the current challenges of congenital microtia with time-consuming, complicated sculpture, limited fabrication material, and fragility, further study is needed prospected to design an optimal cartilage framework to treat microtia with optimum artificial material, great contour appearance, minimal complications, and good enough copy to the natural healthy ear.

## Data Availability

The raw data supporting the conclusions of this article will be made available by the authors, without undue reservation.
