# Supplementary material for: Shape Optimization of Costal Cartilage Framework Fabrication Based on Finite Element Analysis for Reducing Incidence of Auricular Reconstruction Complications
Source: Front Bioeng Biotechnol. 2021 Dec 13;9:766599. doi: 10.3389/fbioe.2021.766599 (PMC8711272; doi:10.3389/fbioe.2021.766599)
Supplement: Supplementary file 1 [file DataSheet1.docx]

***Supplementary material***


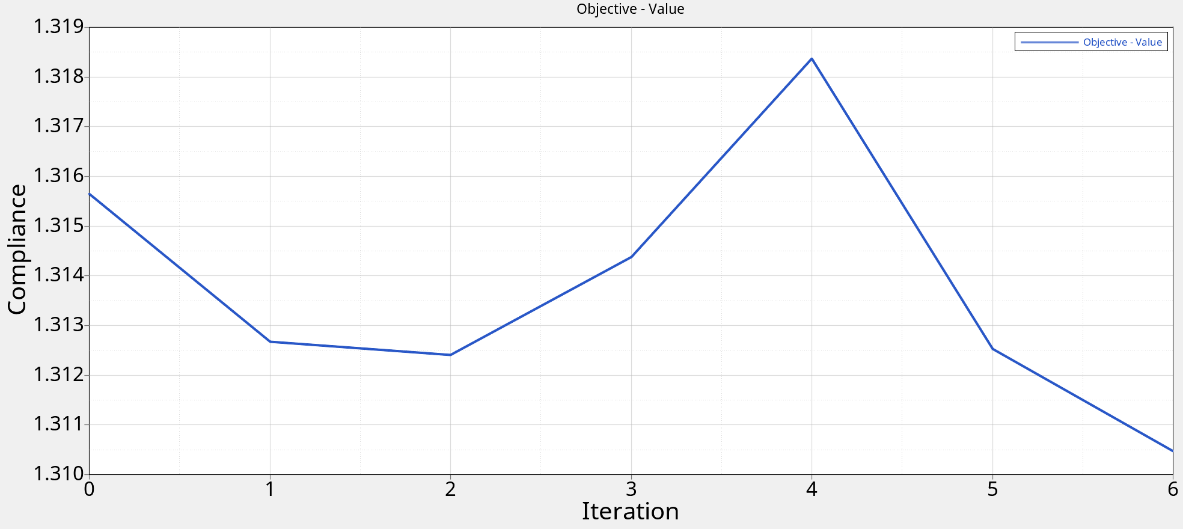


**Figure S1**. Compliance curve vs. iteration numbers for optimization with design variables of shape 1


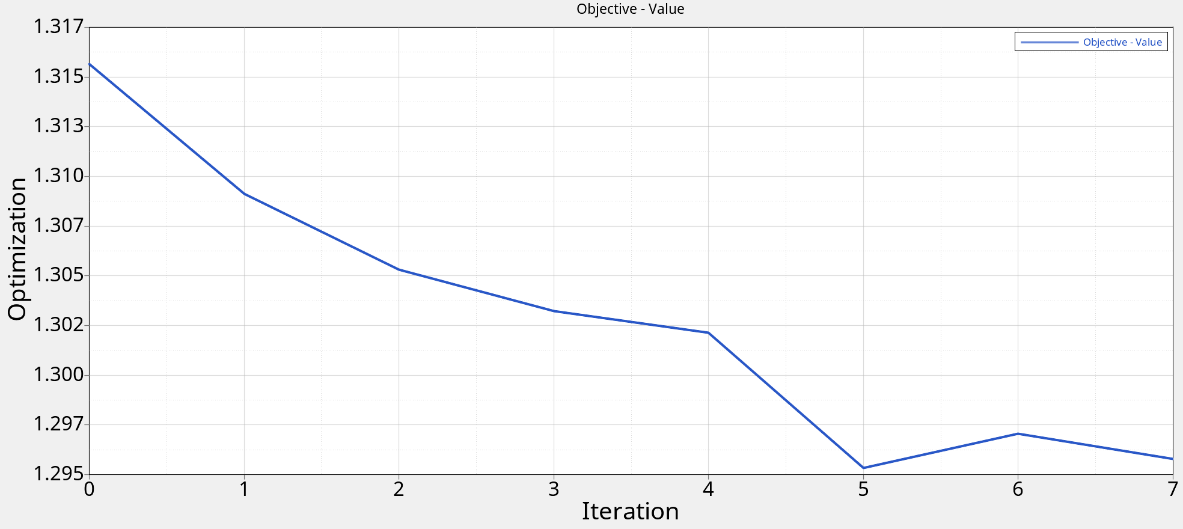


**Figure S2**. Compliance curve vs. iteration numbers for optimization with design variables of shape 2


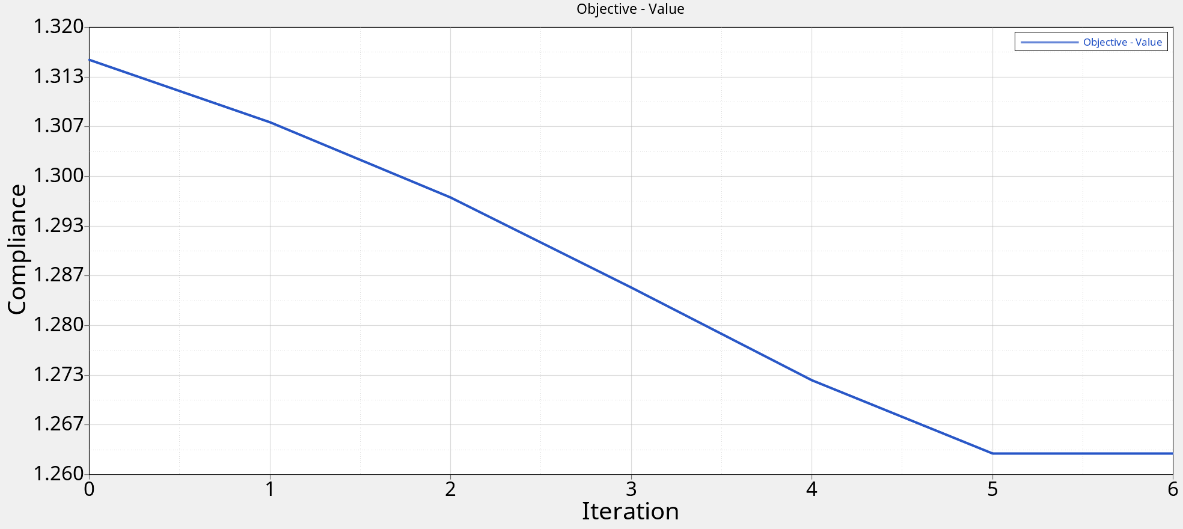


**Figure S3.** Compliance curve vs. iteration numbers for optimization with design variables of shape 3


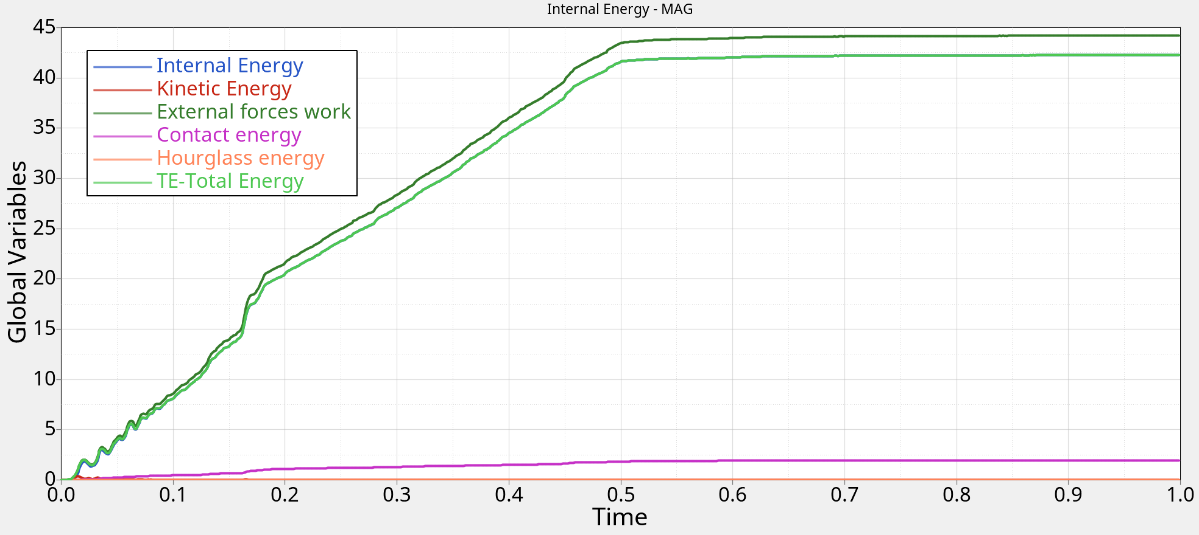


**Figure S4.** Energy conservation results of dynamic finite element simulation
